# Biochemical and Physiological Responses of Harmful *Karenia mikimotoi* to Algicidal Bacterium *Paracoccus homiensis* O-4

**DOI:** 10.3389/fmicb.2021.771381

**Published:** 2021-11-30

**Authors:** Ning Ding, Yanbing Wang, Junfeng Chen, Siyu Man, Feng Lan, Chao Wang, Lijun Hu, Peike Gao, Renjun Wang

**Affiliations:** College of Life Sciences, Qufu Normal University, Qufu, China

**Keywords:** *Karenia mikimotoi*, *Paracoccus homiensis*, algicidal activity, ROS, photosynthesis

## Abstract

Harmful algal blooms caused by *Karenia mikimotoi* frequently occur worldwide and severely threaten the marine environment. In this study, the biochemical and physiological responses of *K. mikimotoi* to the algicidal bacterium *Paracoccus homiensis* O-4 were investigated, and the effects on the levels of reactive oxygen species (ROS), malondialdehyde content, multiple antioxidant systems and metabolites, photosynthetic pigments, and photosynthetic index were examined. The cell-free supernatant in strain O-4 significantly inhibited *K. mikimotoi* cell growth. The bacterium caused the *K. mikimotoi* cells to activate their antioxidant defenses to mitigate ROS, and this effect was accompanied by the upregulation of intracellular antioxidant enzymes and non-enzyme systems. However, the overproduction of ROS induced lipid peroxidation and oxidative damage within *K. mikimotoi* cells, ultimately leading to algal death. In addition, the photosynthetic efficiency of the algal cells was significantly inhibited by O-4 and was accompanied by a reduction in photosynthetic pigments. This study indicates that O-4 inhibits *K. mikimotoi* through excessive oxidative stress and impaired photosynthesis. This research into the biochemical and physiological responses of *K. mikimotoi* to algicidal bacteria provides insights into the prophylaxis and control of harmful algal blooms *via* interactions between harmful algae and algicidal bacteria.

## Introduction

Harmful algal blooms (HABs) are typically related to the discharge of nitrogen and phosphorus nutrients from industry and agriculture and cause considerable threats to fisheries and public health worldwide (Zhang et al., 2014; Berdalet et al., 2016). *Karenia mikimotoi* is a dominant dinoflagellate species in large-scale red tides that causes the mortality of benthic and pelagic organisms by secreting toxic substances (Mooney et al., 2010; Kurekin et al., 2014; O’Boyle et al., 2016; Aoki et al., 2017). Various approaches and techniques (including physical, chemical, and biological methods) have been developed to prevent and control HABs (Lee et al., 2013). Various organisms and their metabolites are potential suppressors of HABs, including algicidal bacteria (Wang et al., 2012), actinomycetes (Zhang et al., 2013), viruses (Cai et al., 2011), and macrophytes (Zhou et al., 2010).

Studies on the interactions between algae and bacteria have resulted in the isolation of algicidal bacteria primarily belonging to the Bacteroidetes, Firmicutes, and Proteobacteria. The representative algicidal bacteria in Bacteroidetes include *Flavobacterium* sp. (Zheng et al., 2018), *Cytophaga*, and *Cellulophaga* (Imai et al., 2006). *Bacillus* (Oh et al., 2011) is the representative algicidal bacteria in the Firmicutes. *Halomonas* (Fang et al., 2012), *Vibrio* (Iwata et al., 2003), *Alteromonas* (Iwata et al., 2003), *Pseudoalteromonas* (Imai et al., 2006), *Thalassospira* (Lu et al., 2016), *Alteromonas* sp., *Marinobacter* sp., *Idiomarina* sp., and *Paracoccus* sp. (Zheng et al., 2018) are the most reported algicidal bacteria in Proteobacteria. *Flavobacteria* sp. is widely found in a number of different environments and exhibits algicidal activity against *Prorocentrum micans* by a direct attack (Shi et al., 2012). *Pseudoalteromonas haloplanktis* AFMB-08041 could suppress the harmful dinoflagellate *Prorocentrum minimum* with an algicidal rate up to 94.5% (Kim et al., 2009). The *Micrococcus luteus* strain SY-13 can secrete an extracellular substance that causes cell lysis in the red tide dinoflagellate *Cochlodinium polykrikoides* (Kim et al., 2008). The genus *Paracoccus* sp. is used in the biodegradation of wastewater treatment and is under investigation for its ability to lyse *Prorocentrum donghaiense* (Zhang et al., 2018).

Algicidal bacteria can inhibit algal growth or lyse algae by attacking the cells directly or indirectly by secreting extracellular substances, including proteins, polypeptides, biosurfactants, amino acids, and antibiotics (Zhuang et al., 2018). The mechanisms involved in the algicide of HABs primarily involve four pathways: cell structure damage, alteration of enzymatic or non-enzymatic systems, inhibition of algal photosynthesis/respiration, and restriction of gene expression (Zhang et al., 2010; Pokrzywinski et al., 2017). To understand the mechanisms of the algicidal process, the physiological and biochemical responses in algal cells require investigation. Aquatic organisms, including algae, can boost their antioxidant defense systems to ease the degree of damage caused by harmful reactive oxygen species (ROS) and lipid peroxidation. Superoxide dismutase (SOD), catalase (CAT), peroxidase (POD), glutathione peroxidase (GPx), glutathione disulfide (GSSG), and macromolecular compounds (such as carotenoids and glutathione) form antioxidant defense systems to prevent damage from the external environment (Yang et al., 2011; Kirilovsky, 2015). Hu et al. (2015) reported that the *Bacillus* sp. Y1 and Y4 decreased photosynthetic pigment content, induced ROS production, and upregulated enzymatic antioxidant systems in HABs.

Previously, we isolated an algicidal bacterium, *Paracoccus homiensis* O-4, and investigated its algicidal activity against *K. mikimotoi*. In the present study, the physiological and biochemical responses of the alga to the algicidal substances from *P. homiensis* O-4 were further investigated particularly from the following aspects: (1) the algicidal mode of *P. homiensis* O-4 against *K. mikimotoi*; (2) the extent of oxidative damage and antioxidant systems activity of algal cells; and (3) the effects of strain O-4 on the algal photosystem in *K. mikimotoi*. Thus, the study objective was to elucidate the biochemical and physiological responses of the algal cells to the algicidal activity of the bacterium and guide the potential application of *P. homiensis* O-4 in controlling HABs dominated by *K. mikimotoi*.

## Materials and Methods

### *Karenia mikimotoi* and Cultivation

*Karenia mikimotoi* was obtained from the Laboratory of Microalgae Research, Ocean University of China, Qingdao, China. The axenic algae were cultured at 25°C in sterile f/2 medium (Lananan et al., 2013) prepared with 0.45-μm filtered natural seawater, with a light intensity of approximately 80 μmol photons m^–2^ s^–1^ under a 12 h:12 h light:dark cycle. The axenic culture of *K. mikimotoi* was tested by culturing on the plates and microscopy method. Cell numbers were counted using a hemocytometer under a light microscope (CX21FS1; Olympus, Tokyo, Japan).

### Algicidal Activity of Strain O-4 on *Karenia mikimotoi*

The strain *P. homiensis* O-4 was previously isolated from seawater (Zheng et al., 2018; Ding et al., 2021). The 16S rRNA gene sequence of the strain was deposited in GenBank (MG457257). Seawater was obtained from Luxun Park, Qingdao, China. The bacterial strain was cultured in 2216E agar medium (peptone 5 g, yeast extraction 1 g, ferric phosphorous acid 0.1 g, agar 10 g, pH 7.6–7.8, fixed capacity to 1 L using sterile seawater) at 25°C for 72 h. For the algicidal test, 1-, 3-, and 5-ml amounts of the bacterial solutions (with volume ratios of 1%, 3%, and 5%, respectively) were each inoculated into the 100-ml flask containing exponentially growing *K. mikimotoi* algal cultures. The algal cells were fixed with Lugol’s iodine. The algicidal activity was monitored by counting the cell numbers using a microscope. Algicidal activity by O-4 was calculated according to the following equation: algicidal activity (%) = (1–*Tt*/*Ct*) × 100%, where *T* and *C* are the concentrations of algal cells in the treatment and control groups, respectively, and *t* is the incubation time. All experiments were performed in triplicate.

### Algicidal Mode of Strain O-4

To assess the mode of action in the algae inhibition of *K. mikimotoi*, the cell-free filtrate was used: the bacterial culture after 72-h cultivation was centrifuged at 15,000 × *g* for 10 min at 4°C and passed through a 0.22-μm membrane filter (Merck Millipore, Darmstadt, Germany). The remaining cell pellets in the bottle were washed twice with a sterile 2216E medium. The cells were resuspended in f/2 medium with the same concentration (3%), shaken, and then labeled as O-4 cells. The control group comprised an algae culture supplemented with a 3% sterile 2216E medium.

### Measurement of Photosynthetic Pigments and Photosynthetic Index

The contents of chlorophyll a (Chl *a*) and carotenoid (Car) were analyzed after the 3 and 5% strain O-4 treatment. Algal cells were collected *via* centrifugation (5,000 rpm, 20 min) after 0, 4, 8, 12, 24, and 48 h of culture. Algal pigments were extracted using 85% acetone solution in the dark at 4°C for 24 h, followed by centrifugation (5,000 rpm, 10 min). The absorbance of the supernatant was measured at the wavelengths of 470, 645, and 663 nm (Gan and Lian, 2021). The absorbance of 85% acetone was used as the control. The formula of photosynthetic pigment is as follows:


Ca(mgL)-1=12.21×A663nm-2.81×A645nm

Cb(mgL)-1=20.13×A645nm-5.03×A663nm

$$(\text{Cc}\left(\mathrm{mg}L^{-1}\right)=\frac{\left(1,000-\mathrm{A470}\,\text{nm}-3.27\,\mathrm{Ca}-104\,\mathrm{Cb}\right)}{229}$$

where Ca, Cb, and Cc represent the concentrations of chlorophyll a, chlorophyll b, and total carotenoid.

The maximum photochemical quantum yield of photosystem II is Fv/Fm, representing the maximum photosynthetic potential. The chlorophyll fluorescence parameters of the treated algae cells were measured using a water pulse amplitude modulation chlorophyll fluorescence analyzer (Walz, Germany). Algal cells were dark-adapted for 15 min before the experiment. The algal fluorescence was detected using a measuring light (0.01 μmol photons m^–2^ s^–1^) with a saturation pulse (0.8 s, 3,500 μmol photons m^–2^ s^–1^). The maximum photochemical quantum yield of the photosystem (Fv/Fm) was used to indicate the efficiency in light energy conversion during photosynthesis.

### Measurement of Reactive Oxygen Species Content

The intracellular ROS level within *K. mikimotoi* was measured using a ROS detection kit (Biyuntian, Shanghai, China) with slight modifications. The methods were as follows: (1) after 4, 8, 12, 24, 36, and 48 h of culture, 40 ml of algal solution was centrifuged at 4°C, and the supernatant was immediately discarded to collect algal cells; (2) DCFH-DA probe dye was added, and the mixture was incubated at 37°C for 30 min; (3) the mixture was centrifuged (at 1,000 rpm, 5 min), and the algal cells were washed with PBS; (4) the mixture was centrifuged again to settle the solution, and the supernatant was discarded to obtain the algal cells; (5) after resuspending the cells using PBS, the fluorescence intensity was measured with an excitation wavelength of 488 nm and an emission wavelength of 525 nm using a flow cytometer (Novocyte 2040R, ACEA, United States).

### Measurement of Malondialdehyde Content and Superoxide Dismutase, Catalase, Peroxidase, and Glutathione Peroxidase Activity

Bacterial filtrates of O-4 were inoculated in the exponential phase axenic *K. mikimotoi* cultures until the concentration of the bacterial solution reached 3 and 5%. An axenic 2216E medium of the same volume was added separately to act as a control. After co-culture for 0, 6, 12, 24, and 48 h, algal cells were collected using centrifugation (10,000 rpm, 20 min), followed by washing with PBS (50 mM, pH 7.4). The cell disruption was assessed using an ultrasonic cell disruption system (200 W, 5 s; 10 s, five times at less than below 4°C). The extracting solution was centrifuged for 15 min at 10,000 × *g*, and the supernatant was used in the cell membrane permeability analysis. Lipid peroxidation levels were measured by assessing the malondialdehyde level following the methods by Dogru et al. (2008).

The crude protease solution was also obtained as described previously (Ding et al., 2018). After co-culture for 0, 6, 12, 24, and 48 h, the algal cell suspension was centrifuged at 4°C for 20 min (10,000 rpm min^–1^). The supernatants were discharged, and the algal cells were collected. The algal cells were washed with PBS (0.05 mol L^–1^, pH 7.8) and transferred to a test tube. Under ice-bath conditions, the algal cells were crushed by ultrasound for 5 min (5 s, interval of 10 s, 200 W). An amount of 1.5 ml of supernatant was absorbed into the Eppendorf tube and centrifuged again at 4°C for 15 min. The supernatants obtained after centrifugation comprised the crude protease solution to be measured. The SOD, CAT, and POD activities in the algal cells were measured following the manufacturer’s instructions (Jiancheng, Nanjing, China). Glutathione peroxidase was measured using a GPx assay kit (Biyuntian, Shanghai, China).

### Measurement of Glutathione, Glutathione Disulfide, Ascorbic Acid, and Dehydroascorbic Acid

Glutathione (GSH) was determined following the instructions in a GSH assay kit (Jiancheng, Nanjing, China). Glutathione oxidized (GSSG) was measured using a GSSG assay kit (Solarbio, Beijing, China). Ascorbic acid (AsA) and dehydroascorbic acid (DHA) were determined *via* the method described in Ding et al. (2018) as follows. Supernatants of 200 μl were prepared (as described above), then 200 μl of dithiothreitol (5 mmol L^–1^) and 500 μl of potassium phosphate of buffer solution (100 mmol L^–1^, pH 7.4) were added to the test tube, and the mixture was incubated at 25°C for 10 min. Then, 100 μl of *N*-ethylmaleimide, 400 μl of trichloroacetic acid (0.61 mol L^–1^), 400 μl of phosphoric acid (0.8 mol L^–1^), 400 μl of 2,2’-bipyridine (0.5 mol L^–1^), and 200 μl of ferric chloride (3 mol L^–1^) were added to the mixture. They were set in the water bath and incubated at 55°C for 10 min. The absorbance of each tube was measured at a wavelength of 525 nm. The total ascorbic acid content could then be calculated. In the above process, the dithiothreitol and *N*-ethylmaleimide were not added, and alternatively, the double steaming water and the reduced ascorbic acid content could be measured. The DHA content was the difference between total AsA and AsA content.

### Data Analysis

The data were presented as means, and their standard errors were analyzed using SPSS 20.0 (SPSS Inc., Chicago, IL, United States).

## Results

### Algicidal Activity and Mode of Strain O-4

**FIGURE 1:**
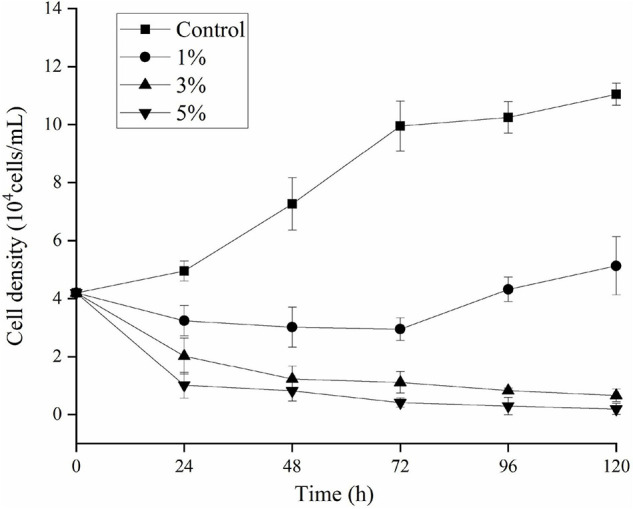
Algicidal activity levels using different doses of *Paracoccus homiensis* O-4 against *Karenia mikimotoi*. Error bars represent the standard deviation of the triplicates.

The effects of O-4 on the growth of *K. mikimotoi* are presented in Figure 1. The results indicate that the algicidal activity by strain O-4 against *K. mikimotoi* cells was concentration-dependent. When compared to the control group, the addition of 1% O-4 exhibited no algicidal activity (*p* > 0.05), and within 120 h, the growth of *K. mikimotoi* slightly increased. In the treatment groups receiving 3 and 5% O-4 bacterial culture, there was significant growth inhibition (*p* < 0.05). The 3% O-4 caused 83% of the cells to be lysed after the treatment duration of 48 h. In the 3% O-4 treatments, 94% of the cells were lysed after 120 h. The 5% bacterial concentration of O-4 exhibited the strongest algicidal activity, leaving no visible intact algal cells after 120 h. The results suggest that the algicidal effects are enhanced with an increased bacterial concentration and treatment duration. Due to the strong algicidal activity in the 3 and 5% O-4 bacterial cultures, they were used in the subsequent stages of our evolving research program.

**FIGURE 2:**
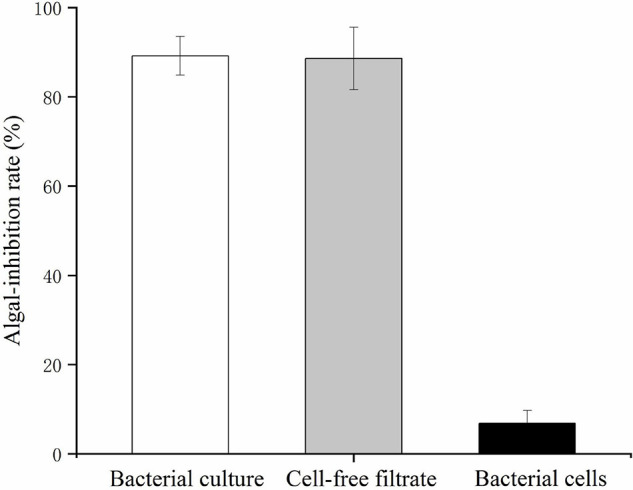
Algicidal activities against *Karenia mikimotoi* by *Paracoccus homiensis* O-4 treatment groups (bacterial culture, cell-free filtrate, and bacterial cells). Error bars represent the standard deviation of the triplicates.

The bacterial cells and cell-free filtrate were separated and inoculated into *K. mikimotoi* cultures to explore the action of the algicidal mode of O-4. The results are provided in Figure 2. The additional bacterial cells did not significantly inhibit the growth of *K. mikimotoi*. However, both the cell-free filtrate and bacterial culture caused a significant inhibitory effect on *K. mikimotoi* (*p* < 0.05). The cell-free filtrate of O-4 reduced the algal cell density, and the algicidal activity against *K. mikimotoi* was 88.6% after 60 h. These results imply that the algicidal activity by O-4 is indirect.

### Effects of Strain O-4 on the Algal Photosystem in *Karenia mikimotoi*

**FIGURE 3:**
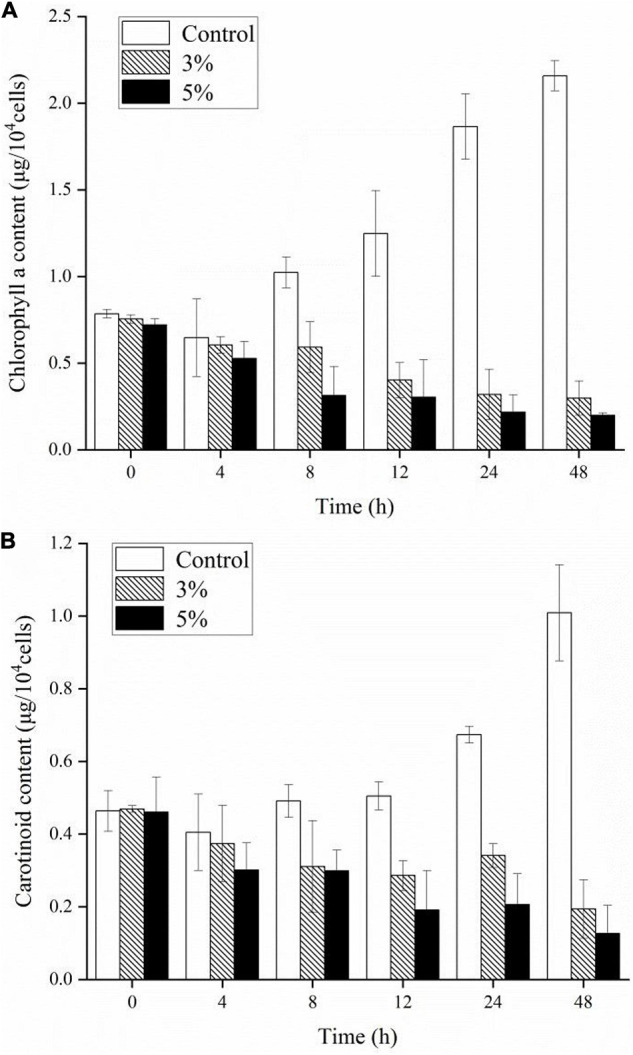
Effects of different doses of *Paracoccus homiensis* O-4 on the Chl *a* **(A)** and carotenoid contents **(B)** in *K. mikimotoi*. Error bars represent the standard deviation of the triplicates.

To investigate the stress caused by O-4 on the algae, we determined the Chl *a*, carotenoid content, and maximum quantum yield of photosystem (PS) II [variable fluorescence (Fv)/maximum fluorescence (Fm)]. Figures 3A,B demonstrate that the pigment contents of the algal cells treated with O-4 were significantly lower than that in the control group after 48 h (*p* < 0.01). After 12 h, the contents of both Chl *a* and carotenoids in the 3% O-4 treatment groups decreased to 32% (Chl *a*) and 57% (carotenoids), respectively, compared to the control. After 48 h, the pigment content was reduced by approximately 86% (Chl *a*) and 60% (carotenoid) compared to the control. Moreover, after 48 h, the reduction in Chl *a* and carotenoid contents in the 5% O-4 treatment groups was approximately 90% (Chl *a*) and 91% (carotenoids) with respect to the control. These results indicate that O-4 is capable of damaging pigments in algal cells.

**FIGURE 4:**
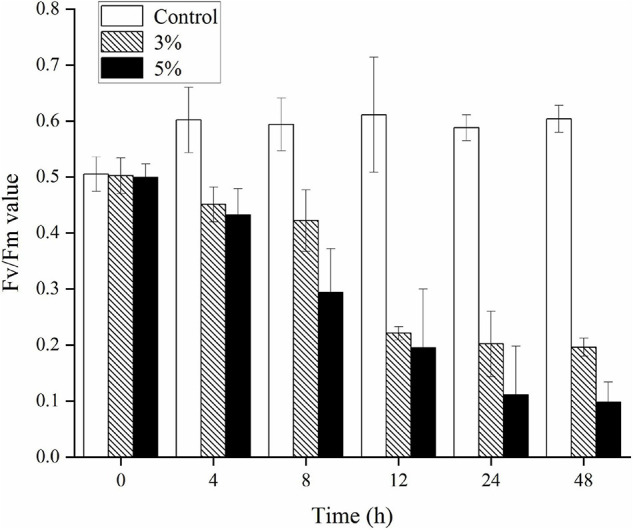
Effects of photosynthetic efficiency (Fv/Fm) within *Karenia mikimotoi* treated with varying doses of *Paracoccus homiensis* O-4. Error bars represent the standard deviation of the triplicates.

The Fv/Fm ratio was evaluated to determine the photosynthetic status of the algal cells after the 3 and 5% O-4 treatments (Figure 4). The Fv/Fm values decreased significantly relative to untreated cells after 12 h (*p* < 0.05). As the treatment duration progressed, the Fv/Fm values continued to decrease. At 48 h, when compared to the Fv/Fm values of the control group, the 3% treatment group was 3.1-fold lower (*p* < 0.05), and the 5% treatment group was 6.2-fold lower (*p* < 0.01. These results demonstrate that the photosynthetic capacity was inhibited in the treated cells (Figure 4).

### Reactive Oxygen Species Levels and Lipid Peroxidation of *Karenia mikimotoi* Under Algicidal Activity

**FIGURE 5:**
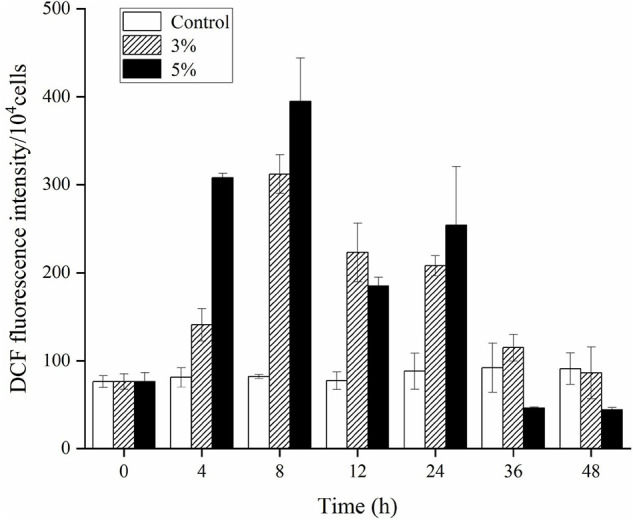
Effects of ROS levels within *Karenia mikimotoi* after exposure to different doses of *Paracoccus homiensis* O-4. Error bars represent the standard deviation of the triplicates.

An ROS level analysis explored the oxidative stress caused by O-4 in *K. mikimotoi* cells. Figure 5 shows a slight increase in DCF fluorescence intensity in the control group. Conversely, the DCF fluorescence intensity was significantly increased in algal cells treated with O-4 (*p* < 0.05, Figure 5). The ROS levels were significantly increased after 8 h of exposure in both treatment groups containing O-4, with ROS levels 3.8-fold (3% O-4) and 4.8-fold (5% O-4) higher than the control group. However, the ROS levels in both treatment groups began decreasing after 12 h of exposure, and at 48 h, the ROS content of algal cells in both treatment groups was maintained at a low level compared to the control.

**FIGURE 6:**
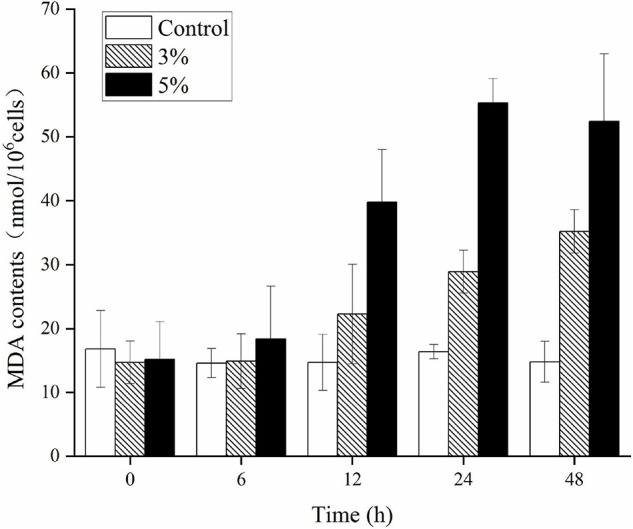
Effects of MDA contents in *Karenia mikimotoi* after exposure to different volumes of *Paracoccus homiensis* O-4. Error bars represent the standard deviation of the triplicates.

Figure 6 illustrates the effects of O-4 on lipid peroxidation in the algal cells. Algae cells exposed to O-4 exhibited a pronounced increase in MDA content. The MDA levels in all the treated groups were higher than those in the controls (*p* < 0.05), and MDA content increased with exposure duration and increased concentrations of O-4. At 48 h, the MDA levels were 2.4 (3% O-4 group) and 3.5 (5% O-4 group) times higher than the control group.

### Responses of Antioxidative Enzymes to Strain O-4 Treatment

**FIGURE 7:**
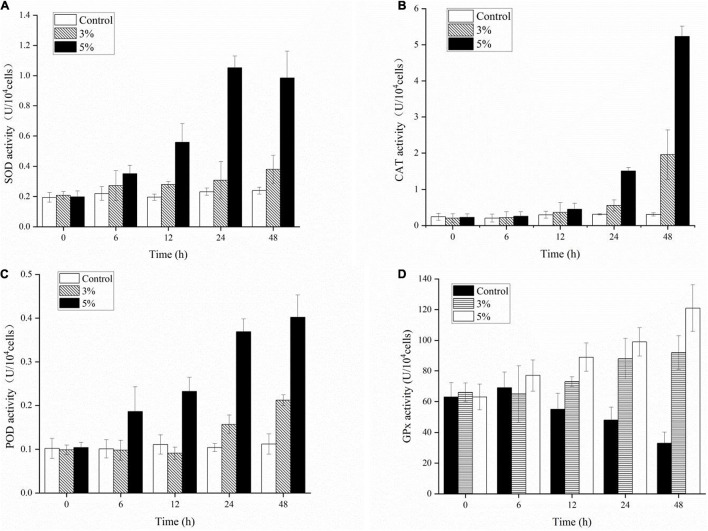
Effects of enzymic antioxidants including SOD **(A)**, CAT **(B)**, POD **(C)**, and GPx **(D)** activities in *Karenia mikimotoi* after exposure to varying levels of *Paracoccus homiensis* O-4.

We investigated the physiological defense responses induced by exposure to the algicidal O-4 by determining the representative enzymatic activities within cells, including SOD, CAT, POD, and GPx (Figures 7A–D). Figure 7A demonstrates that SOD activity increased as the exposure duration increased relative to the control in all treatment groups (*p* < 0.05). The SOD activity initially decreased slightly until 48 h of exposure in the 5% group. The CAT values were significantly higher in the 3% O-4 treatment groups when compared to the control (Figure 7B); the values were 1.2-fold higher after 12 h, 1.78-fold after 24 h, and 6.36-fold after 48 h (*p* < 0.05). The CAT values were significantly higher in the 5% O-4 treatment groups when compared to the control; the values were 1.51-fold higher (*p* < 0.05) after 12 h, 4.82-fold (*p* < 0.05) after 24 h, and 17.0-fold after 48 h (*p* < 0.01). The POD values of 3% O-4 treatment group were slightly lower than the control groups after 6-h and 12-h exposure but increased significantly with exposure duration and additional ratios (Figure 7C), reaching a peak at 48 h (*p* < 0.05). The GPx activity had similar results to the POD activity (Figure 7D). When algal cells were exposed to 3 and 5% O-4 bacterial cultures for 48 h, the activity values were 2.79 and 3.67 times higher than the control, respectively (*p* < 0.05).

### Responses of Antioxidative Non-enzymes to Strain O-4 Treatment

**FIGURE 8:**
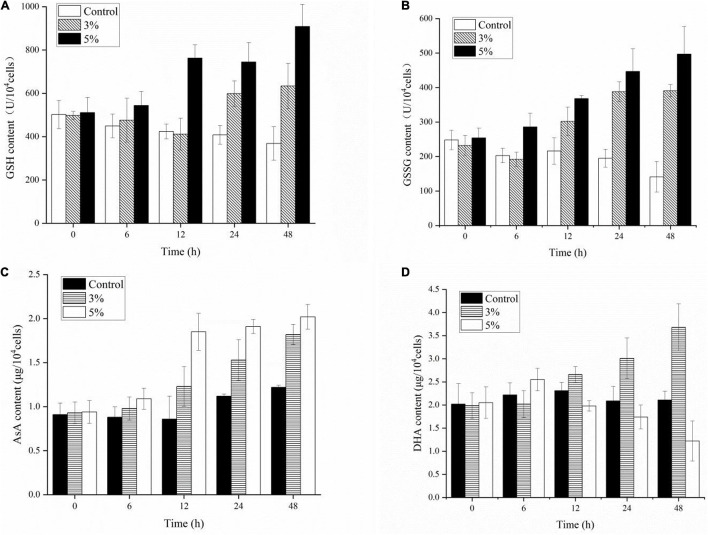
Effects of non-enzymic antioxidants including GSH **(A)**, GSSG **(B)**, AsA **(C)**, and DHA **(D)** activities in *Karenia mikimotoi* after exposure to *Paracoccus homiensis* O-4 at different doses.

Antioxidant non-enzymatic activities (including GSH, GSSG, AsA, and DHA) were assayed to analyze the algal cell protective responses against O-4 (Figures 8A–D). Figure 8A shows that GSH was stimulated by O-4 (*p* < 0.05). The only exception was the 3% treatment group after 12 h. The GSH content in the treatment groups compared to the control groups increased from 498 to 634 μmol L^–1^ (3%) and 511 to 909 μmol L^–1^ (5%) within 48 h. The GSSG contents of the algae in both treatment groups increased significantly with increasing O-4 concentrations and treatment duration and remained higher than the control group over 48 h (*p* < 0.05, Figure 8B). The AsA levels in *K. mikimotoi* cells were similar to the GSSG results (Figure 8C). At 48 h, the AsA levels were 1.5-fold (3% O-4) and 1.66-fold (5% O-4) higher than the control group. Figure 8D graphically demonstrates the effects of the strain O-4 on DHA in *K. mikimotoi* cells. After 12 h, the 3% O-4 group exhibited a stimulatory effect on DHA. Within 48 h, there was a significant inhibitory effect on the DHA in the 5% O-4 treatment group (*p* < 0.05).

## Discussion

Over the past decades, HABs have frequently occurred in eutrophic coastal areas of Europe and China, and *K. mikimotoi* is a dominant HAB species (Qian et al., 2009; Kurekin et al., 2014). Some marine bacteria can promote or reduce algal blooms (Teeling et al., 2012). Algicidal bacteria can act as potential biological controllers in the degradation and termination of HAB species (Schoemann et al., 2005). In this study, we evaluated the algicidal activity and inhibitory mechanisms of the *P. homiensis* strain O-4 against the harmful *K. mikimotoi*. The algicidal activity of O-4 was time- and concentration-dependent, using 3 and 5% ratios of bacterial culture in approximately 90 and 95% algal lysis over 96 h, respectively. The density of algal cells in high concentrations of the bacterial culture (3 and 5%) decreased significantly compared to the control and 1% concentration groups, which suggested that strain O-4 had an effective algicidal activity on *K. mikimotoi*, and the higher concentrations were even more efficient. Thus, the results suggest that this strain is a viable algal-lysing agent for regulating *K. mikimotoi*. A similar study revealed a marine algicidal bacterium *Mangrovimonas yunxiaonensis* strain LY01 caused 87% inhibition of toxic *Alexandrium tamarense* after 60 h of exposure (Li et al., 2014).

We explored the biochemical and physiological responses of *K. mikimotoi* induced by the algicidal bacterium *P. homiensis* O-4. A direct attack requires direct contact between the bacteria and algal cells, whereas an indirect attack occurs when algicidal bacteria produce algicidal substances to inhibit algal cells and does not require cell-to-cell contact. The addition of cell-free filtrate and bacterial culture showed algicidal activity. Conversely, the washed bacterial cells exhibited no significant algicidal effect on *K. mikimotoi*. Therefore, the O-4 algicidal mode was indirect, as the algicidal activity was likely to be expressed through the excretion of extracellular algicidal substances. Our results were consistent with a previous study that found that the algicidal bacteria *Hahella* sp. KA22 can lyse the toxic dinoflagellate *Heterosigma akashiwo* by producing the extracellular compound prodigiosin (Zhang et al., 2020).

Direct and indirect external stress can induce algal cells to trigger excessive ROS levels, which cause severe oxidative damage or cellular death (Apel and Hirt, 2004). ROS, including O^–2^, O_2_, HO_2_, H_2_O_2_, RO, OH, ROO, and ROOH, are relatively reactive in live cells and are continuously generated as the by-products of diverse metabolic pathways in various cellular organelles, such as the mitochondria, chloroplasts, and peroxisomes. Consequently, the proteins, DNA, lipids, and carbohydrates of cells are damaged by excessive ROS, eventually leading to cell death (Gill and Tuteja, 2010). In the present study, O-4 caused an ROS explosion in *K. mikimotoi* cells compared to the controls within a short exposure (8 h in all treatment groups).

These results imply that the stress caused by O-4 induced algal cells to produce excess ROS, ultimately causing cell death. The ROS caused oxidative damage to *K. mikimotoi* cells, as evidenced by the upregulation of the MDA content within cells upon exposure to O-4. Malondialdehyde is considered an indicator of lipid peroxidation and is a major peroxidation product that reflects the degree of cellular oxidative damage (Yamauchi et al., 2008). Cell membranes consist of primarily unsaturated phospholipids and are sensitive to oxidative attack; therefore, additional ROS results in excess accumulation of MDA (Qian et al., 2008). The increase in MDA levels within algal cells after exposure to O-4 indicated that the O-4 induced membrane lipid peroxidation and caused oxidative damage to the cell membrane systems of *K. mikimotoi.* This phenomenon has previously been observed, resulting in the MDA content being upregulated after a short exposure to an allelochemical (Qian et al., 2009).

To protect living cells from oxidative damage and environmental stress, important enzymatic antioxidants are engaged, including superoxide dismutase (SOD), catalase (CAT), peroxidase (POD), and glutathione peroxidase. Superoxide dismutase and POD can catalyze the dismutation of O^–2^ to H_2_O_2_ and O_2_, and degradation converts them into H_2_O and O_2_ under the promotion of CAT and GPx, easing the impact of oxidative stress and initiating cell repair (Valentine et al., 1998; Kwok et al., 2012). Additionally, to reduce oxidative stress induced by algicidal compounds, a series of non-enzymatic antioxidants (including AsA, GSH, and GSSG) is also activated to scavenge the excess intracellular ROS. The O^–2^ and H_2_O_2_ can also be removed by AsA to reduce the damage from lipid peroxidation (Deutsch, 1998). As antioxidants, GSH and GSSG play many important roles in modulating the redox environment of the membrane and cell-wall-related proteins and maintain the sulfur status to protect cells from various stresses (Aziz et al., 1996). Our results suggest that antioxidant enzymes and non-enzyme systems were initiated at varying levels after exposure to O-4. The algicidal compounds generated by O-4 were toxic to the *K. mikimotoi* cells and caused them to produce excessive ROS. The antioxidant systems may be responsible for the algicidal activity in O-4 by strengthening the activities of both antioxidant enzymes and non-enzyme systems. However, high doses of O-4 were fatal to *K. mikimotoi* cells because the high ROS levels surpassed the capacity of the cells to defend themselves, ultimately causing cell death. Our results showed that a significant inhibitory effect on DHA occurred when the cells were treated with O-4. Dehydroascorbic acid acts as an important oxidoreductase during metabolic processes, and is relatively sensitive to environmental stress and noxious substances (Zhang et al., 2016). In this study, the algal cell viability was closely associated with DHA. The downregulation of DHA levels, when exposed to O-4, demonstrated that the regular metabolism in *K. mikimotoi* cells was disrupted and crucial enzyme activity was suppressed.

*K. mikimotoi* is a dominant species in marine photoautotrophic phytoplankton, and photosynthesis in algal cells plays a key role in global primary production. The PSII system acts as a major pigment–protein complex that can catalyze photosynthesis and is sensitive to adverse environmental conditions (Nymark et al., 2009). Photosynthetic pigments primarily include Chl *a* and carotenoids in the thylakoid membrane, which harvest light and energy for conversion in the photosynthetic process. The significant declines in Chl *a* and carotenoid contents after exposure to O-4 may be caused by their impacts on electron flow and the regular operation of the PSII system. Another photosynthesis index, Fv/Fm, represents the maximum efficiency of PSII. However, external environmental factors, including light intensity, temperature, and biotic stress, typically decrease the Fv/Fm values (Kumar et al., 2014). The inhibition of Fv/Fm values after treatment with O-4 suggests that the photosynthetic efficiency was seriously impeded and that dysfunction occurred in the PSII system. Overall, the reductions in pigment content and Fv/Fm values, and the transferring of excitation energy to ROS as singlet oxygen, eventually caused a decline in the interference capacity of ROS generation.

In conclusion, the algicidal bacterium *P. homiensis* O-4 exerted an efficient inhibitory effect on *K. mikimotoi* cells. O-4 caused the algae to produce excessive ROS, and the antioxidant enzyme systems increased. The antioxidant non-enzymic substances played a synergistic role in reducing the damage caused by ROS. Meanwhile, membrane lipid oxidation increased, and cell membrane integrity was lost. Photosynthetic systems, including photosynthetic pigments and photosynthetic efficiency, were seriously damaged. Superfluous ROS overloaded the antioxidant defense systems, and damage to critical systems and functions ultimately caused algal cell death.

## Data Availability Statement

Publicly available datasets were analyzed in this study. This data can be found here: GenBank (MG457257).

## Author Contributions

PG and RW conceived and proposed the idea. ND, YW, JC, SM, FL, CW, and LH carried out the experiments and conducted data analysis. ND and PG drafted the manuscript. All authors have read and approved the final manuscript.

## Conflict of Interest

The authors declare that the research was conducted in the absence of any commercial or financial relationships that could be construed as a potential conflict of interest.

## Publisher’s Note

All claims expressed in this article are solely those of the authors and do not necessarily represent those of their affiliated organizations, or those of the publisher, the editors and the reviewers. Any product that may be evaluated in this article, or claim that may be made by its manufacturer, is not guaranteed or endorsed by the publisher.
